# An Easy Route to Wettability Changes of Polyethylene Terephthalate–Silicon Oxide Substrate Films for High Barrier Applications, Surface-Modified with a Self-Assembled Monolayer of Fluoroalkylsilanes

**DOI:** 10.3390/polym11020257

**Published:** 2019-02-03

**Authors:** Paola Scarfato, Nicola Schiavone, Gabriella Rossi, Loredana Incarnato

**Affiliations:** 1 Department of Industrial Engineering, University of Salerno, Via Giovanni Paolo II, 132-84084 Fisciano (SA), Italy; grosssi@unisa.it (G.R.); lincarnato@unisa.it (L.I.); 2 Université Clermont Auvergne, CNRS, SIGMA Clermont, ICCF F-63000 Clermont–Ferrand, France; nicola.schiavone@etu.uca.fr

**Keywords:** self-assembled monolayer (SAM), fluoroalkylsilanes, nanocoating, wettability, atomic force microscopy

## Abstract

Inorganic–organic multilayer films consisting of polymers coated with thin inorganic oxidic layers (e.g., SiOx) ensure very high barrier performances against gas and vapor permeation, what makes them packaging materials suitable for sophisticated technical applications, including the encapsulation of photovoltaic devices or quantum dots, barrier films for optical displays, and transparent greenhouse screens. In these fields, surface coating or texturing of the multilayer protective films are effective technologies to improve their self-clean ability, thus reducing the required maintenance and ensuring longer durability and better performances. In this work, we used the self-assembled monolayer (SAM) technique to modify the surface and wetting properties of commercial polyethylene terephthalate-silicon oxide substrate (PET-SiOx) films developed for technical applications requiring a combined high barrier and transparency. The selected surface modifier was the 1H,1H,2H,2H-per-fluorodecyltrichlorosilane (FDTS). The reagent mixture composition was optimized for the lowest water and oil wettability, as well as the highest self-cleaning capacity and performance stability. In particular, for the used PET-SiOx film the best FDTS/film surface for both the lowest water and oil wettability was found to be equal to 26.5 mM/dm^2^, which changes the surface behavior from very hydrophilic (static water contact angle (CAw) = 21.5°) to hydrophobic (CAw = 101°), and gives a significant increment of the static oil contact angle (CAo) from 27° to 60°. Interestingly, the results demonstrated that the SAM reaction occurred also on the uncoated the PET side. After the SAM treatment, a small increase of the water vapor permeability is observed, probably due to a crack or defect onset of the SiOx coating of the SAM modified films. On this point, atomic force measurements demonstrated an increment of the SiOx coating layer roughness after the SAM treatment execution. Finally, the transparency changes of the SAM treated films, measured in the wavelength range 400–800 nm, were always small, so that the results were acceptable for the films’ use in applications where high transparency is required.

## 1. Introduction

Nowadays, for a variety of high-performance industrial applications, including microfluidic devices, controllable drug delivery systems, self-cleaning surfaces, films and screens for solar cells, optical displays, and greenhouse coverings, it is important to control the wetting behavior of liquids on the device surfaces, in order to have lubrication and water-repellent coatings. Liquid behavior on a surface can range from complete spreading, as in the “tears of wine” effect, to minimal wetting, as observed on a superhydrophobic lotus leaf [1]. In particular, the devices developed for photovoltaic and optical applications, to retain high efficiency of all their components during their lifetime, require complex packaging solutions consisting of several layers, each of which have a specific functionality, such as acting as a moisture and liquid barrier, self-cleaning, anti-reflection and thermal radiation properties, flexibility, transparency, etc. [2,3,4,5]. The combination in one layer more functionalities can offer the possibility of simplifying the packaging structure, and thus its manufacturing cost, ensuring also high efficiency and durability. Therefore, many research and technological efforts are devoted in pursuing this aim by means of different strategies (e.g., mechanical treatments, exposure to flames or corona discharge, wet-chemical treatments, etc.) able to modify the properties of the encapsulating layers, specially the polymeric ones [6,7,8,9,10,11,12,13,14,15,16,17].

In particular, a lot of interest has been devoted to improving the polymer film surfaces’ wettability for obtaining hydrophobic and oleophobic surfaces through the self-assembled monolayers (SAM) technique for surface functionalization [2,18,19,20]. This technique has been demonstrated to be effective in creating protective nano-coatings on silicized high-barrier polymer substrates, such as PET-SiOx films, by the facile deposition of highly hydrophobic silane precursor molecules (alkylsilanes and (fluoro)alkylsilanes) on the SiOx layer of the substrate. However, despite the simplicity of the SAM technique, the formation of a reproducibly well-defined monolayer is exceedingly difficult [21,22]. One of the main issues with this system is the casual formation of polycondensed silane (i.e., multilayered products) or physically adsorbed silane molecules on the SiOx surface, particularly when no activation step to promote the grafting is used. Furthermore, the deposition process is especially complicated; it proceeds through a number of stages and strongly depends on various parameters, including solvent and adsorber concentrations, silanol density at the active surface, cleaning procedures, aging of solutions, water content, deposition time, and temperature. Initial reports claimed water traces to be essential for the formation of well-packed monolayers. Recent studies have shown that the increase in water content or age of the adsorbed solution promotes island-type growth. To further complicate the debate, the film growth seems to proceed by different mechanisms under “dry” or “wet” conditions [23].

In this context, we are carrying out a study aimed to develop a simple route to change the wettability of PET-SiOx films for high barrier applications. In previous works, we developed a method to obtain flexible and transparent hydrophobic SAM nanocoating using different alkyl- and fluoroalkylsilanes as precursor molecules, by means of simple and effective single-step process carried out at room temperature and applied to a polyethylene terephthalate–silicon oxide substrate (PET-SiOx), with the demonstrated efficacy of SAM in improving the oxygen and water barrier characteristics of commercial PET-SiOx films, normally used in photovoltaics, without losing significant transparency and assessing high durability of the developed systems in aggressive environments [2,24,25]. In this work, using as precursor molecule the best among those already tested, i.e., 1H,1H,2H,2H-per-fluorodecyltrichlorosilane (FDTS), we intended to optimize the conditions of the deposition reaction to minimize the film wettability. To this aim, the SAM reaction was carried out using different compositions of the FDTS reagent mixture, in order to evaluate the effect on the water and oil wettability of the modified PET-SiOx films and select the optimal reaction conditions for the lowest wettability. Optical transparency and the surface roughness have also been measured.

## 2. Material and Methods

### 2.1. Materials

The polymeric substrate used for the experiments was a flexible and transparent silicon oxide (SiOx)-coated polyester film provided by AMCOR (Zürich, Switzerland), consisting of a 12 μm thick polyethylene therephthalate (PET) coated by a 50 nm thick SiOx layer deposited by electron beam evaporation. The molecule used as precursor for the SAM deposition was 1H,1H,2H,2H-per-fluorodecyl-trichlorosilane 96% (FDTS; C_10_H_4_Cl_3_F_17_Si, MW 581.65, *d* = 1.540 g/cm^3^), provided by Alfa Aesar (Lancashire, UK), and the solvent was anhydrous ethanol (EtOH, ≤ 0.01% water), provided by Sigma Aldrich (Milan, Italy). All the chemicals were used as purchased without further purification.

### 2.2. Deposition Experiments for the Preparation of Nanocoated Samples

**Figure 1:**
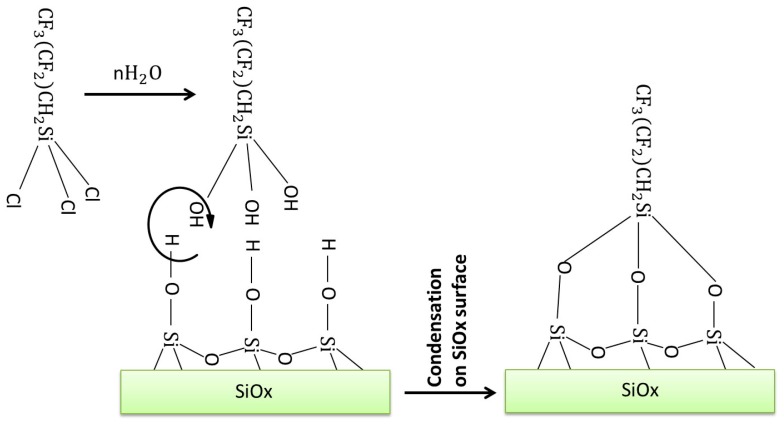
Sketch of the reaction between the 1H,1H,2H,2H-per-fluorodecyltrichlorosilane (FDTS) and the polyethylene terephthalate–silicon oxide substrate (PET-SiOx) film.

Before the functionalization experiments, the PET-SiOx substrate films were accurately cleaned in order to remove all the impurities from their surfaces. The cleaning procedure consisted of three consecutive cycles of soaking in ethanol for 5 min, drying in a nitrogen flow, and rinsing in distilled water, with a final drying in a nitrogen flow. The deposition experiments were performed at 25 °C in a glass batch reactor, preliminarily flame-dried in order to remove any trace of water. Subsequently, an N_2_ atmosphere was maintained in the reactor. The silane was deposited under anhydrous conditions for 24 h, submerging the substrate films (having surface area equal to 1.00 dm^2^) in ethanolic solutions containing different volume amounts of FDTS, thereby realizing different values of silane amount per film surface area. The reaction is sketched in Figure 1.

The functionalized samples were then cleaned by three consecutive cycles of soaking in ethanol and distilled water, with intermediate drying in N_2_ flow.

In order to investigate possible substrate modifications due to a functionalization procedure instead of a silane reaction, samples consisting of PET–SiOx films submitted to all the steps of the deposition process without the use of FDTS were also prepared, for comparison purposes. The list of the produced samples and their nomenclature are reported in Table 1.

**Figure 2:**
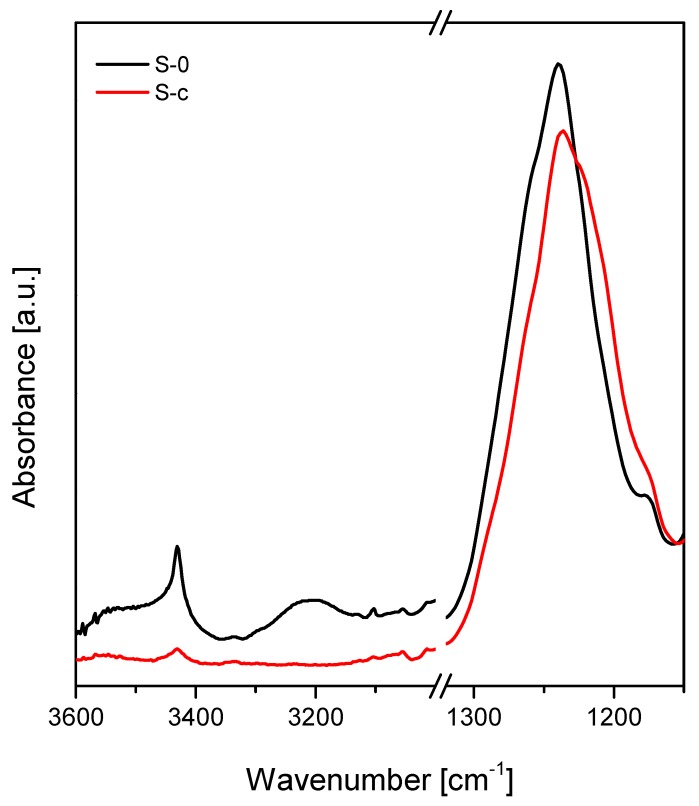
Fourier transform infrared spectroscopy in attenuated total reflection mode (ATR-FTIR) spectra of PET-SiOx film before (S-0) and after (S-c) FDTS condensation.

In order to verify that in the adopted experimental conditions, the FDTS condensation reaction on the PET–SiOx substrate films successfully occurred, film samples before and after deposition were submitted to Fourier transform infrared (FTIR) spectroscopy analysis, according to procedure described in Section 2.3. The FTIR spectra of the S-0 (uncoated) and S-c (coated) samples evidenced changes in spectral regions of the Si–OH bands (3600–3100 cm^−1^) and of the broad Si–O band (1300–1100 cm^−1^), as reported in Figure 2. The graph shows that in the S-c curve the Si–OH bands almost disappear, because during deposition experiments they combine with FDTS to form new Si–O–Si linkages. At the same time, a new feature in the region of the Si–O band appears, consisting of a shoulder at approximately 1220 cm^−1^, which is attributed to the Si–C linkages of the FDTS fluoroalkylsilane chains condensed on the PET-SiOx substrate [2,26].

### 2.3. Methods 

Fourier transform infrared spectroscopy measurements in attenuated total reflection mode (ATR-FTIR) were carried out in the 4000–650 cm^−1^ spectral range, using a Nexus ThermoNicolet spectrometer (Madison, WI, USA) equipped with a SmartPerformer accessory.

Static contact angle measurements were performed with a First Ten Angstrom Analyzer System 32.0 mod. FTA 1000 (First Ten Angstroms, Inc., Portsmouth, VA, USA), according to the standard test method ASTM D5946, using distilled water and synthetic polyalphaolefin base oil PAO6 as test liquids. The drop volume was taken within the range where the contact angle did not change with the variation of the volume (2 ± 0.5 μL). Each reported value of the θ angle is the average of five replicate measurements.

Water vapor permeability tests were performed with a water vapor permeation analyzer Model 7002 (Systech Illinois, UK) that uses a sensor of P_2_O_5_. The measurements were carried out at 23 °C and a relative humidity of 85%, according to the standard ASTM F 1249. The standard deviations of all measured water vapor transmission rate (WVTR) values were <5%.

The surface morphology was characterized using a Nanoscope V multimode atomic force microscope (AFM) (Digital Instruments, Bresso, Italy), and the root-mean-square roughness (Rq) values were calculated on scan areas of 5 μm × 5 μm with the Software Nanoscope Analysis of Bruker (version 1.40).

Dynamic mechanical analyses (DMA) were carried out using a Q800 analyzer (TA Instruments, New Castle, DE, USA). The tests were performed in tensile mode, at a frequency of 1 Hz, in the temperature range of 30–190 °C, under a heating rate of 3 °C/min.

Transparency measurements were performed on the film samples using a Lambda 800 UV– VIS Spectrophotometer (Perkin-Elmer Inc., Waltham, MA, USA), according to ASTM D1746-03. The spectra were recorded at a scan speed of 2 nm/s in the wavelength range 800–200 nm. 

## 3. Results and Discussion

**Figure 3:**
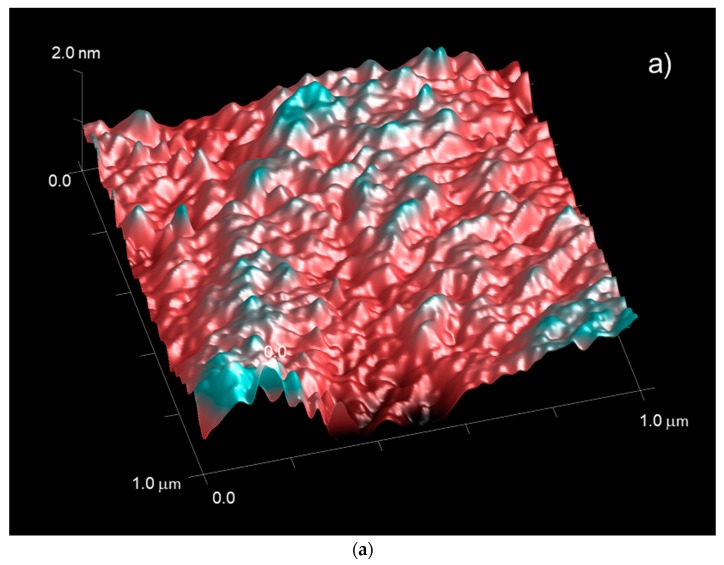

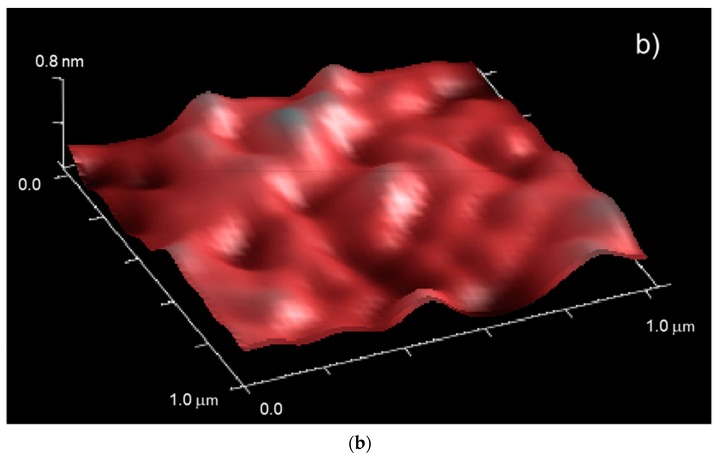
Three-dimensional (3D) atomic force microscope (AFM) images for the original untreated PET-SiOx film: (**a**) uncoated PET side and (**b**) SiOx-coated side.

In order to assess the surface properties of the original untreated PET-SiOx substrate, its water and oil wettability and its roughness were measured on both sides of the film (i.e., the uncoated PET side and the SiOxcoated side) by static contact angle and atomic force microscopy measurements. The results were reported in Table 2 and Figure 3. 

**Figure 4:**
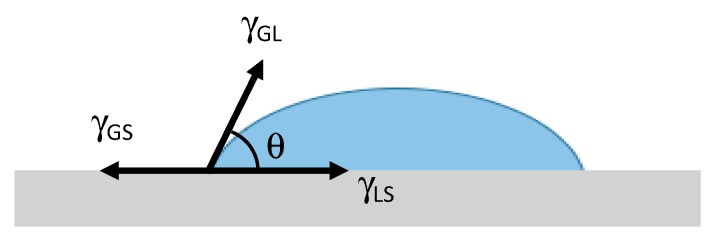
Illustration of the static contact angle θ, formed by sessile liquid drops on a solid surface, and of the relative surface energies of solid–liquid (SL), gas–liquid (GL), and gas–solid (GS).

As expected, the SiOx coating strongly increases the surface water wettability of the film, as it comes out comparing the CAw values of the two sides of the substrate. The phenomenon is due to the higher polarity of the SiOx coating layer, with respect to the uncoated PET, which allows favorable Coulomb interactions (i.e., interactions among permanent–permanent dipoles, e.g., hydrogen bonds and interactions among permanent-induced dipoles) with the water droplets. On the other hand, almost no effect on the oil wettability was detected, since both sides of the films have similar CAo values, maybe due to the very low surface tension of the oil (27.5 mN/m at 25 °C [27]. It is well known, in fact, that the static contact angle *θ* is a function of the relative surface energies of the solid–liquid (SL), gas–liquid (GL), and gas–solid (GS), as shown in Figure 4. 

This relationship is mathematically described by the Young equation: *γ*_GS_ = *γ*_LS_ + *γ*_GL_cos*θ*. In the case of complete wetting (spreading), the contact angle is 0°. This means that the solid sample has a much high surface energy than the liquid; between 0° and 90°, the solid substrate is wettable, and above 90° it is not wettable. This means that the solid has a surface energy much lower than the liquid. 

In terms of surface texture, which strongly influences the wetting behavior of a surface [28], AFM analyses demonstrated that the SiOx coating layer is quite homogeneous and smooth, and has much lower surface roughness, in the range of atomic sizes, than the PET side of the film. 

**Figure 5:**
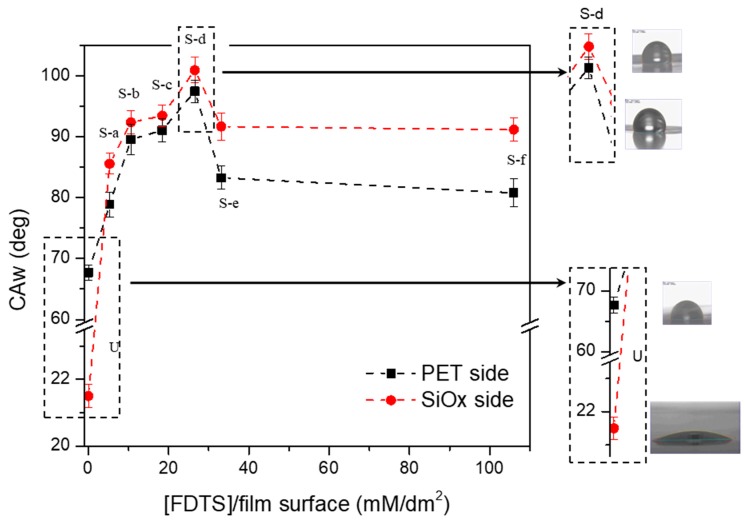
Effect of the reaction conditions on the water contact angles (CAw) of the films: black square marks indicate the uncoated PET side, and the red circle marks indicate the SiOx-coated side.

**Figure 6:**
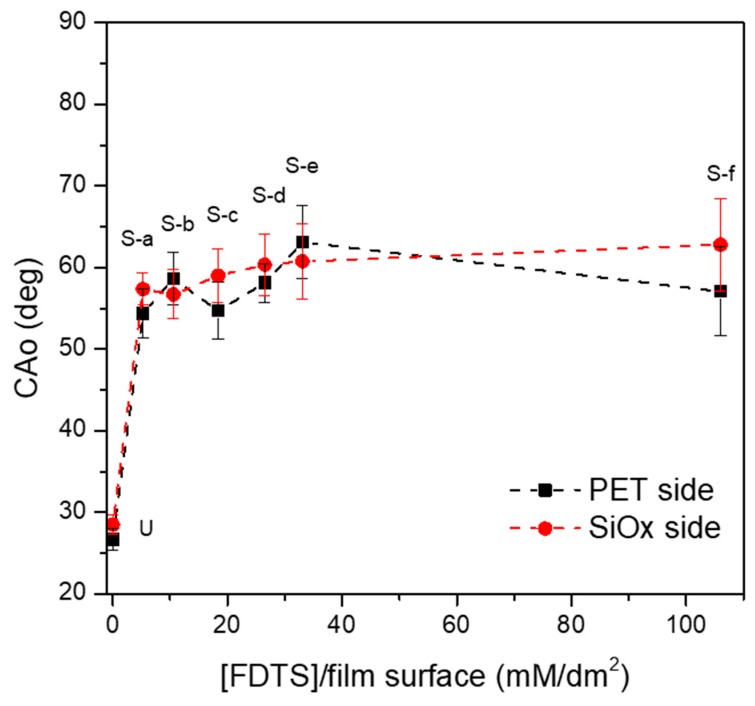
Effect of the reaction conditions on the oil contact angles (CAo) of the films: black square marks indicate the uncoated PET side, and the red circle marks SiOx-coated side.

With the aim of analyzing the effect of the silane concentration per film surface area on the changes in film wettability, due to SAM reaction, all the modified samples were submitted to static contact angle measurements, using water and oil as a test liquid. Figure 5 and Figure 6 compare the CAw and CAo values, respectively, evaluated on both sides (i.e., the uncoated and the SiOx-coated side) of the films.

The graph reported in Figure 5 shows that the SAM treatment significantly increased the CAw values, not only for the SiOx-coated side, but also, unexpectedly, for the uncoated one. The increase is particularly relevant on the SiOx coating layer, whose behavior changes from very hydrophilic (CAw = 21.5°) to hydrophobic (CAw > 90°), with a gain of CAw ranging in the interval of 65–80° depending on the reaction conditions, and a maximum value of CAw equal to 101° measured for the S-d sample. The same trend was measured also on the uncoated PET side of the substrate, with CAw values only slightly lower with respect to the SiOx-coated side, and a maximum equal to 97.5° for the S-d sample. Of course, the gain of CAw measured on the PET side of the film is always smaller in this case, ranging in the interval of 10–30°, since the starting CAw value of the uncoated PET side is already high (CAw = 67.7°).

A similar evolution of the static contact angle was also observed with oil as test liquid, as shown in Figure 6. However, in this case the CAo values show more statistical fluctuations. This result is coherent with what is reported in the literature: Yang et al. have obtained several correlations between different surface substrates and contact angles for crude oil, demonstrating that this measurement is strongly dependent on oil properties [29].

**Figure 7:**
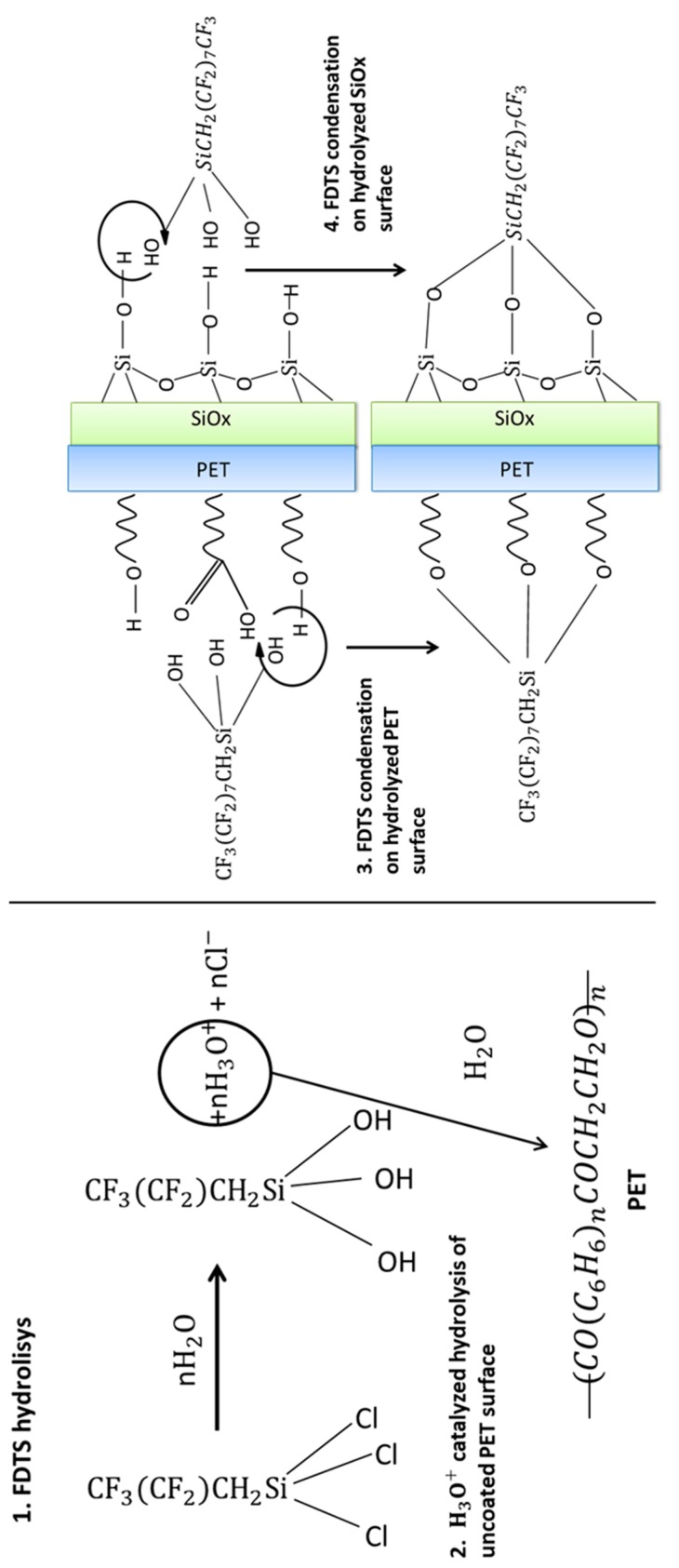
Sketch of the possible reaction mechanism between the FDTS and the uncoated PET layer of the PET-SiOx film.

The water and oil wettability changes of the uncoated film surface suggests that, in the adopted experimental conditions, FDTS is also able to give the condensation reaction with the hydroxyl groups of PET molecules. A possible reaction mechanism, involving a step in which the PET chains are activated by acid hydrolysis, is schematically depicted in Figure 7 [17,30].

In order to investigate if the SAM treatment, together with the reduced wettability, was effective in decreasing the moisture transport properties of the film, water vapor permeability measurements were carried out. The results, listed in Table 3, show that all treated films have WVTR values about five-fold higher than the original untreated substrate, independent from the reaction conditions. Since the high barrier against moisture is provided by the SiOx coating layer, it may be hypothesized that, in the adopted experimental conditions, the manipulation operations related to the SAM process execution, rather than to the SAM reaction itself, produce a deterioration of the coating layer integrity. 

With the aim of verifying this hypothesis, the films S-0 and S-d, chosen as examples, were tested by AFM and DMA analyses. The AFM gave information on the surface roughness and quality of the SiOx coating layer, whereas DMA is a very sensitive technique for detecting the dynamic mechanical behavior of polymers at the molecular level and their main thermal transitions, providing information on the film’s morphology.

**Figure 8:**
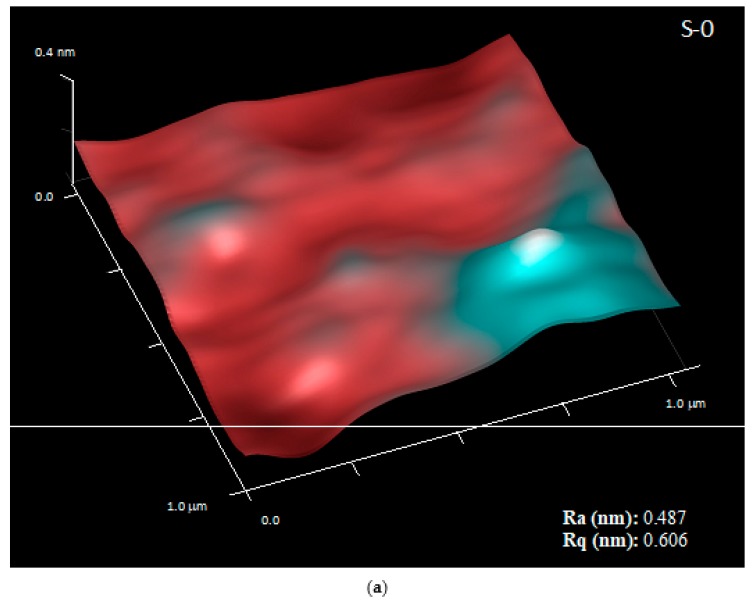

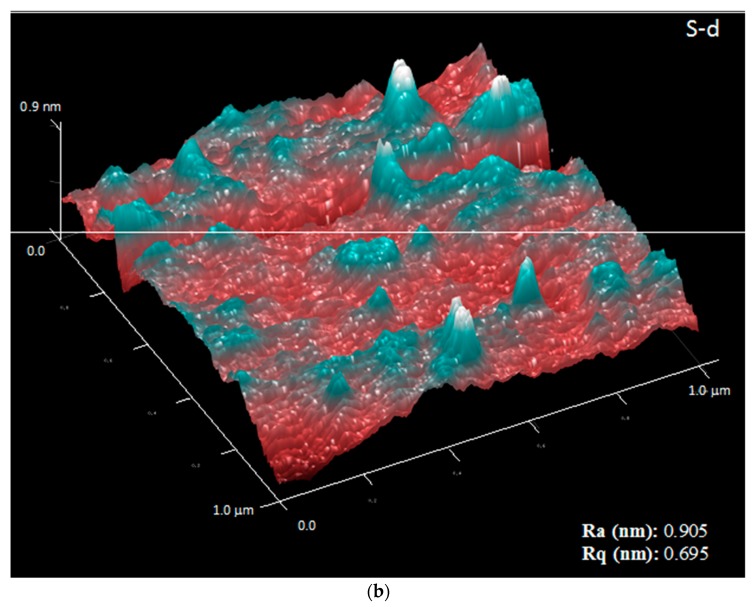
AFM images with scan area taken on the SiOx-coated side of S-0 (**a**) and S-d (**b**) films, and corresponding values of arithmetic average roughness (Ra) and root-mean square roughness (Rq).

The results of AFM measurements, reported in Figure 8, show that the adopted experimental procedure for SAM treatment execution altered the SiOx coating layer, significantly increasing its roughness with respect to the original substrate. However, it is interesting to note that the effects are similar with and without FDTS, indicating that the most critical point of the procedure lies in the handling operations of the film rather than in the reaction with the silane molecule [31].

**Figure 9:**
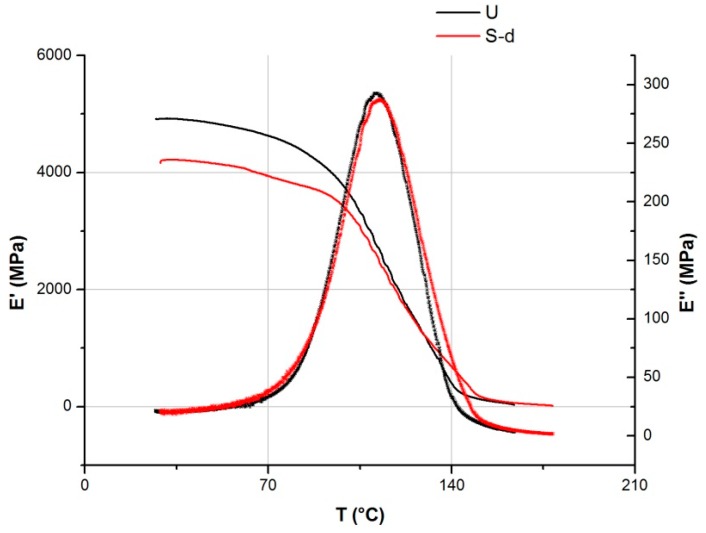
DMA curves of PET-SiOx film before (black) and after (red) the SAM process execution.

As a consequence of this change, as it comes out from DMA curves reported in Figure 9, a reduction of the storage modulus E’ of the film was also observed, associated with the film’s “stiffness” in the low temperature range where the material is in the glassy state. However, the Tg value, measured as the peak temperature of the loss modulus E’’ curve, remained unchanged. This indicates that the SAM treatment has not altered the morphological state of the polymer, but only affected the SiOx coating layer. This result also highlights the need for the SiOx coating layer to be protected from possible physical–mechanical actions that can damage its integrity and functionality.

**Figure 10:**
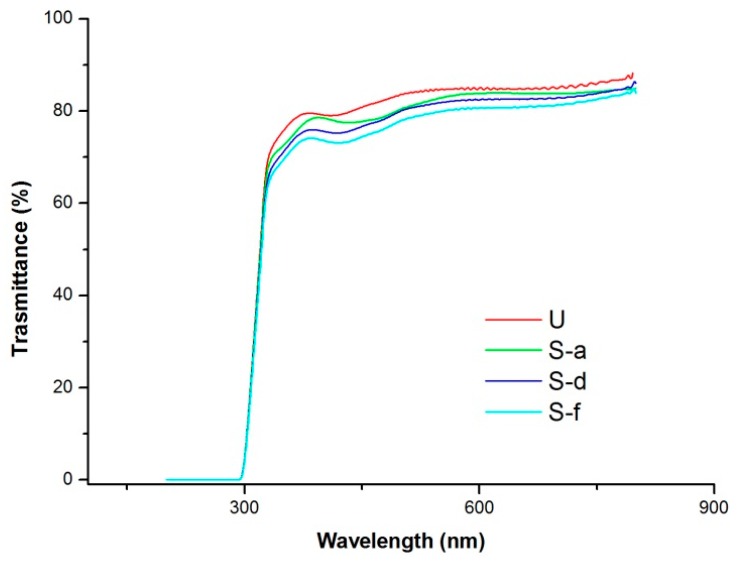
Effect of the reaction conditions on the % transmittance in the UV–visible range of the untreated and some SAM =0treated PET-SiOx films.

In order to evaluate the effects of the SAM treatment on sample transparency, a critical parameter for possible application of the used PET-SiOx film in technical fields where optical properties are crucial (e.g., photovoltaics, optical displays, greenhouse screens, etc.), all treated systems were submitted to UV-Vis spectroscopic analyses. The obtained UV-Vis transmittance spectra, reported in Figure 10, underlines that the original PET-SiOx substrate shows a high transparency, close to 86% in the wavelength range 400–800 nm. This transparency slightly decreases after SAM treatment up to a value of 80% for the S-d film; however, it still remains acceptable for the application areas of interest.

## 4. Conclusions

In this work, the self-assembled monolayer technique was used as a tool for surface and wetting property modification of commercial PET-SiOx films, suitable for high-performance technical applications in power, electronics, and construction fields. The SAM reaction was carried out using different compositions of the reagent mixture, in order to evaluate the effect on the water and oil wettability of the modified films, and to select the optimal reaction conditions for the lowest wettability, as well as the best self-cleaning capacity and performance stability. In particular, for the PET-SiOx film used, the best FDTS/film surface ratio for both the lowest water and oil wettability was found equal to 26.5 mM/dm^2^, which changes the surface behavior from very hydrophilic (CAw = 21.5°) to hydrophobic (CAw = 101°), and gives a significant increment of the CAo from 27° to 60°. Interestingly, the results demonstrated that the SAM reaction also occurred on the uncoated layer of the PET-SiOx substrate; therefore, it is able to reduce, even if for a minor entity, the film wettability on the PET side, too. The wettability reduction is accompanied by a small increase of the water vapor permeability, probably due to a cracks or defect onset of the SiOx coating of the SAM modified films. On this point, AFM analyses demonstrated an increment of the SiOx coating layer roughness after the SAM treatment execution. Finally, the transmittance lowering of the SAM treated films, measured in the wavelength range 400–800 nm, is always small and acceptable for their use in high-barrier applications where high transparency is required.

## Figures and Tables

**Table 1:** Sample description and nomenclature.

Sample Name	Sample Description	Silane Solution Composition		FDTS/Film Surface (mM/dm^2^)
		EtOH (mL)	FDTS (mL)	
U	Untreated PET-SiOx	0	0	0
S-0	PET-SiOx treated with FDTS in different reaction conditions	100	0	0
S-a		99.80	0.20	5.30
S-b		99.60	0.40	10.6
S-c		99.30	0.70	18.4
S-d		99.00	1.00	26.5
S-e		98.75	1.25	33.1
S-f		96.00	4.00	106

**Table 2:** Static water and oil contact angle (CAw and CAo, respectively), and film roughness.

Sample	CAw (deg)	CAo (deg)	Ra (nm)	Rq (nm)
Uncoated PET side	67.7 ± 0.9	28.5 ± 5.9	1.14	1.39
SiOx side	21.5 ± 0.9	26.6 ± 2.3	0.305	0.373

CAw: water contact angle; Cao: oil contact angle; Ra: Roughness Average (i.e., arithmetic average of the absolute values of the roughness profile ordinates; Rq: Root Mean Square Roughness (i.e., root mean square average of the roughness profile ordinates).

**Table 3:** Effect of self-assembled monolayer (SAM) reaction conditions on the water vapor transmission rate (WVTR) of the films.

Sample Name	WVTR (g·m^−2^·d^−1^)
U	0.39 ± 0.04
S-a	1.89 ± 0.14
S-b	1.93 ± 0.11
S-c	2.22 ± 0.18
S-d	2.10 ± 0.15
S-e	2.00 ± 0.16
S-f	1.91 ± 0.14
